# Stereotactic Magnetic Resonance-guided Online Adaptive Radiotherapy for Oligometastatic Breast Cancer: A Case Report

**DOI:** 10.7759/cureus.2368

**Published:** 2018-03-26

**Authors:** Marguerite Tyran, Minsong Cao, Ann C Raldow, Audrey Dang, James Lamb, Daniel A Low, Michael L. Steinberg, Percy Lee

**Affiliations:** 1 Department of Radiation Oncology, University of California, Los Angeles, Los Angeles, USA; 2 Department of Radiation Oncology, University of California, Los Angeles

**Keywords:** breast, oligometastasis, case report, stereotactic body radiation therapy, magnetic-resonance-guided-radiation-therapy

## Abstract

We present a case of durable local control achieved in a patient treated with stereotactic magnetic resonance-guided adaptive radiation therapy (SMART) for an abdominal lymph node in the setting of oligometastatic breast cancer. A 50-year-old woman with a history of triple positive metastatic invasive ductal carcinoma of the left breast, stage IV (T3N2M1), underwent neoadjuvant chemotherapy, mastectomy, adjuvant radiotherapy and maintenance hormonal treatment with HER2 targeted therapies. At 20 months after definitive treatment of her primary, imaging showed an isolated progressive enlargement of lymph nodes between hepatic segment V/IVB and the neck of the pancreas. Radiofrequency ablation was considered, however, this approach was decided not to be optimal due to the proximity to stomach, and pancreatic duct. The patient was treated with SMART for 40 Gray in 5 fractions. Two and a half years later, the patient remains without evidence of disease progression. She experienced Grade 2 acute and late toxicity that was successfully managed with medications. This experience shows that SMART is a feasible and effective treatment to control the abdominal oligometastatic disease for breast cancer.

## Introduction

Systemic therapy is the standard of care for patients with metastatic breast cancer (BC). However, few patients who achieve complete remission after systemic therapy will maintain a long-term response; speaking for the addition of local treatments for a subset of patients with oligometastatic disease. Performing surgical resection or ablative local treatments such as radiation therapy (RT) or radiofrequency ablation (RFA) is a growing practice in the management of oligometastatic disease for various cancer types. Breast histology, long disease-free interval (>6 months), one to three small metastases and good performance status (Eastern Cooperative Oncology Group (ECOG) 0–1) appear to be the most relevant prognostic factors for evaluating who may benefit from stereotactic body radiation therapy (SBRT) [1].

SBRT is widely used for the treatment of extracranial BC oligometastases [2]. However, some localizations are challenging: visualization and positional uncertainties and/or proximity of gastrointestinal structures traditionally limit the use of ablative doses of radiotherapy. Magnetic resonance-guided-radiation therapy (MR-guided-RT) allows for superior soft tissue definition enabling online adaptive plans in response to inter-fraction organ motion. Additionally, intra-fraction motion can be managed with treatment gating by soft-tissue tracking [3]. MR-guided-RT provides daily personalized treatments because a new adapted plan can be generated for each fraction after reoptimization based on daily set-up and internal anatomy changes. These advanced features may be especially advantageous in abdominal disease sites.

We report a case of oligometastatic BC with an isolated residual abdominal lymph node (LN) treated using stereotactic magnetic resonance-guided adaptive radiation therapy (SMART).

## Case presentation

A 50-years-old woman felt a mass in her left breast in November 2012 confirmed by mammogram and breast ultrasound. She was otherwise healthy without any remarkable past medical or surgical history. A biopsy revealed invasive ductal carcinoma, triple-positive (estrogen receptors 90%, progesterone receptors 85% and her-2 gene amplification). Magnetic resonance imaging (MRI) of the bilateral breasts showed a 4 x 2.3 x 5.5 cm mass extending to the pectoralis muscle. There were multiple axillary LNs whose pathology was consistent with the breast primary.

Systemic staging workup included a normal brain MRI and a positron emission tomography-computed tomography (PET-CT). PET-CT showed the breast fluorodeoxyglucose (FDG) avid mass and multiple lesions in the left axilla. It also revealed an FDG-avid mass in segment IVA of the liver and several LNs in the periportal region and between hepatic segment V/IVB and the neck of the pancreas. The liver biopsy was consistent with the breast primary. She was staged as stage IV (T3N2M1).

She received neoadjuvant chemotherapy with a combination of Docetaxel (five cycles), Herceptin, Pertuzumab, and Tamoxifen. Chemotherapy was stopped due to neurotoxicity (hand neuropathy) and evaluation by PET-CT in April 2013 showed near complete response of her tumor burden with only a residual FDG avidity in the solitary liver metastasis. She underwent RFA to the liver lesion in May 2013. She underwent mastectomy with sentinel LN dissection and oophorectomy in November 2013 revealing 1.5 cm of viable disease, fully resected with clear margins, two involved sentinel nodes with extracapsular extension and negative bilateral ovarian pathology. She then proceeded with chest-wall and regional nodal RT (50.4 Gy/28 fractions completed on 01/24/2014). Subsequently, she was followed with alternating liver-MRI and PET-CT every three months while pursuing Herceptin, Pertuzumab, and hormonal therapy. Surveillance imaging showed an area of low PET-CT positivity between hepatic segment V/IVB and the neck of the pancreas which remained stable and was correlated to slowly growing LNs on the repeated MRI. No other evidence of distant disease was noted.

**Figure 1:**
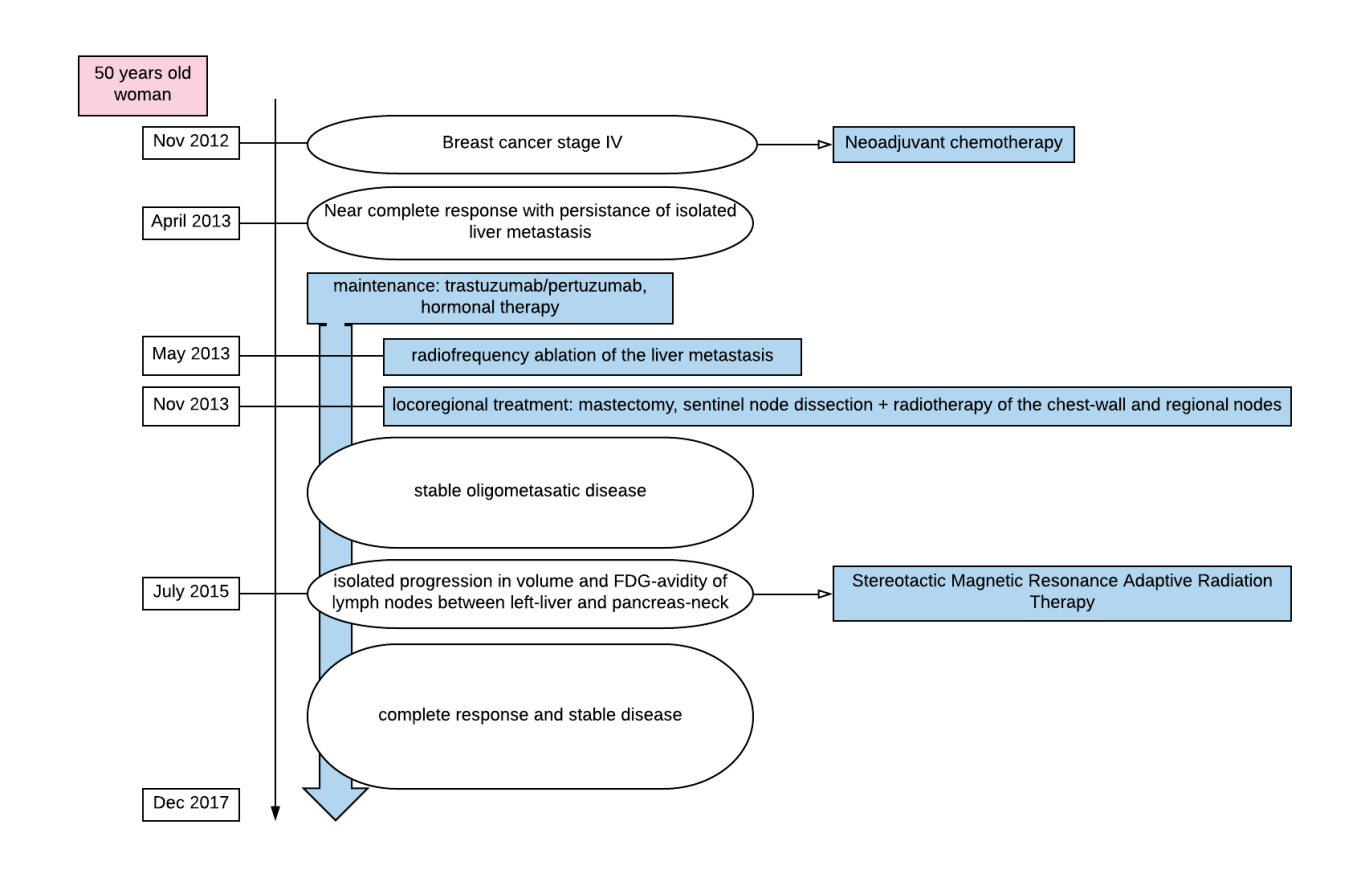
Flow-Chart summarizing patient’s cancer history. FDG: Fluorodeoxyglucose

In July 2015, LNs between hepatic segment V/IVB and the pancreas measured 1.5 cm on the abdominal-MRI. A progressive enlargement of small LNs in the periportal region was also noted but without FDG-avidity. The concomitant PET-CT showed an increased maximum standardized-uptake-value of 18.7 of the hypermetabolic activity adjacent to segment V/IVB of the liver (previously 7.8 in March 2015). Due to her disease being broadly systemically controlled with well-tolerated maintenance systemic therapy with Herceptin, Pertuzumab, and hormonal-therapy, she was proposed for a local treatment of her isolated progressing oligometastasis. RFA was considered, however, due to proximity to stomach, and pancreatic duct, was decided not to be optimal. Thus, she was referred for radiation therapy. Her performance status at that time was ECOG = 0 and she continued to work part-time (Figure 1).

**Figure 2:**
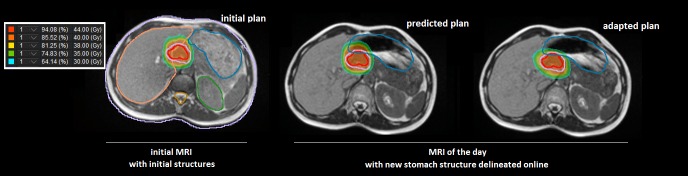
Contours and dose distributions for initial, predicted and adapted treatment plans for the first fraction. Stomach (Blue), gross tumor volume (red) and planning target volume (purple) are displayed on all plans. Position and filling of the stomach on the daily image were very different from initial plan. The predicted plan shows the dose distribution of the initial plan calculated on the current anatomy with updated contours, resulting in excessive dose to the stomach. The adapted plan was re-optimized to avoid excessive dose to the stomach. Isodoses color-lines are displayed in the left box. MRI: Magnetic resonance imaging; Gy: Gray

**Figure 3:**
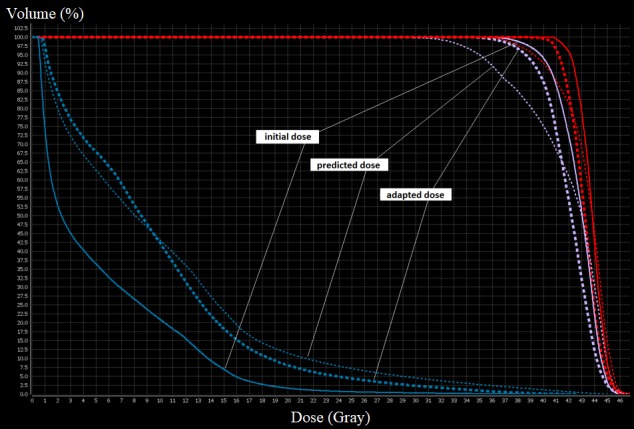
Dose-volume histogram for stomach (blue line), GTV (red line) and PTV (purple line). Predicted plan (dotted line) showed increased dose to the stomach and undercoverage of target volumes (volume of the stomach receiving 35 Gy (V35-stomach) = 8.9 cc, volume of the 95% of the PTV receiving prescribed dose (V40-PTV) = 75%, volume of the GTV receiving 95% of prescribed dose (V40-GTV) = 92.54%) compared with the initial plan (solid line). Adapted plan (dashed line) allows for lower dose to the stomach while restoring coverage of target volumes (V35-stomach = 0.53 cc, V40-PTV = 88%, V40-GTV = 99.54%). GTV: Gross tumor volume; PTV: Planning target volume.

Simulation scans were performed on both a standard CT and a commercial available Tri-Cobalt-60 MRI-guided-RT platform (ViewRay, Inc., Cleveland, OH, USA). Diagnostic MRI and PET-CT were registered with planning MRI and used to assist the delineation of the gross tumor volume (GTV). The planning target volume (PTV) was defined as a 5 mm volumetric expansion of the GTV. The prescribed dose was 40 Gray (Gy)/5 fractions, with the goal of 95% of the volume of PTV covered by the prescription dose. Treatment planning was performed using the dedicated treatment planning system (TPS) (ViewRay MRIdian® system) using five gantry groups, each consisting of three independent multileaf collimator modulated beams. Prior to each treatment fraction a daily set-up MRI was acquired using the same acquisition protocol as simulation. The initial planning contours were transferred from simulation MRI to the daily set-up MRI through deformable image registration between planning and daily setup MRIs, and were reviewed and manually adjusted by the physician and the medical physicist. The initial treatment plan was then re-calculated based on the patient’s current anatomy to ascertain whether the treatment plan was optimal for that day’s specific anatomy. The decision to adapt relied on target coverage (Volume of the PTV receiving the prescribed dose (PTV-V40)) and institutional organs-at-risk (OAR) constraints (Volume receiving 35 Gy (V35)) on the adjacent critical structures: stomach, small bowel, and duodenum. During the daily online adaptive process, we observed modifications of the volume and position of the surrounding OAR which led to the decision to adapt the plan. These modifications led to degraded coverage of the target volume due to its relative position to the OAR; the lesion was intra-peritoneal and not fixed. The volume of the stomach was lowered by 64.5% (42-75.5) on average. Adaptive planning was deemed necessary for all five treatment fractions due to consistent excessive dose to the stomach. The fractions 3 and 5 predicted dose also indicated excessive dose to the duodenum. On-line re-optimization with the original beam angles and optimization objectives was performed for every fraction to create a new adapted plan used for treatment. All adapted treatments were delivered with real-time MRI-guidance including cine-MRI gating on the GTV. Figure 2 shows a representative case of inter-fractional variation of the stomach which led to online adapted planning for this particular treatment fraction. Dose-volume histograms for the initial, predicted and adapted plans are displayed in Figure 3.

No acute toxicity was noted during the course of treatment. However, the patient developed burning pain in her epigastrium several weeks after completion of treatment. She was prescribed omeprazole 20 mg daily which mitigated her symptoms significantly. Due to incomplete relief, she underwent esophagogastroduodenoscopy in June of 2016 revealing a non-bleeding channel ulcer for which omeprazole 40 mg twice daily was prescribed for 10 months (Terminology Criteria for Adverse Events (CTCAE) Grade 2). Dose of the medication was then decreased as her discomfort gradually improved over months. Pyloric location and chronology of the ulcer are suggestive of an adverse event due to RT. Published dose-constraints for SBRT predict a risk of toxicity Grade 3 or greater; the observed Grade 2 which was successfully managed with oral medication was acceptable toxicity in the setting of treating a tumor lesion. No other long-term toxicity was noted. She did not experience any emesis, nausea, diarrhea or disrupted hepatic function at any point.

**Figure 4:**
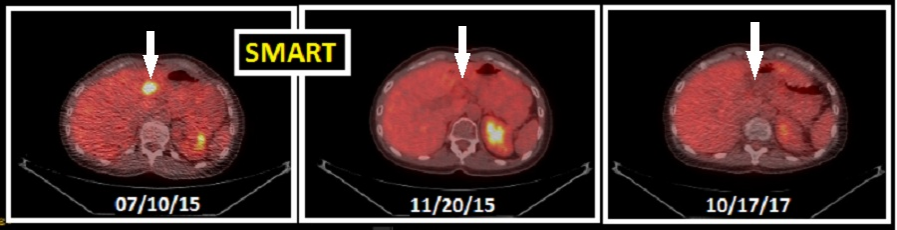
PET-CT pre- and post-treatment with stereotactic magnetic resonance-guided adaptive radiation therapy. Pre-therapeutic imaging on 07/10/15 shows a hypermetabolic activity adjacent to segment V/IVB of the liver which was absent on early evaluation after treatment on 11/20/15. The response was maintained till then as shown on her last imaging on 10/17/17. PET-CT: Positron emission tomography-computed tomography.

For response and disease control assessment, she was closely followed with repeated MRI, PET-CT and labs. Her first PET-CT three months after SMART in November 2015 showed a complete response at the treated site, confirmed by the subsequent surveillance imaging (Figure 4).

Her last evaluation in October 2017 showed overall stable of disease under Herceptin, Pertuzumab, and Fulvestrant. She remained asymptomatic, ECOG = 0, without weight loss on her last visit in December 2017. Her labs showed a remaining low CA 15.3 (18 U/ML), asymptomatic chronic anemia (Hemoglobin = 10.2 g/dl), normal albumin (4.2 g/dl) and preserved hepatic function.

## Discussion

This patient fits the definition of oligometastatic disease which is generally considered by the presence of one to five slower-growing metastases involving a limited number of organs, and that may represent the only vestige of cancer [4]. Our patient exhibits disease control more than five years after receiving her first-line treatment for metastatic BC. She also pursues anti-HER2 therapies which improve survival in HER2-positive metastatic BC patients. The two-year progression-free survival (PFS) reported in a recently published randomized trial was approximately 40% for those patients treated with the combination of Pertuzumab, Trastuzumab, and Docetaxel [5]. However, the LN region treated with SMART was growing under these therapies, demonstrating the utility of combined approaches in order to achieve a durable response and a potential cure for patients.

To our knowledge, this is the first report describing outcomes at two and a half years after SMART for an abdominal oligometastasis of a triple-positive BC. Twenty patients have been treated in a phase I trial using SMART (50 Gy/5 fractions) for oligometastatic or unresectable primary malignancies of the abdomen with a median follow-up of 15 months [6]. No acute toxicities over Grade 3 were observed. One Grade 2 asymptomatic gastric ulcer was noted. They included three para-aortic LNs but results were not detailed for this small subset of patients. Among 11 patients with oligometastatic disease, one-year overall survival was 91% and one-year systemic PFS was 45%. At last follow-up 8/11 were alive without late toxicity nor progression of the systemic disease.

The benefit/risk balance of aggressive local treatments for oligometastases in BC patients should be carefully considered given the lack of randomized data. Risks of SBRT largely correlate with the site of targeted disease and the dose of radiation, and may be reduced when using SMART.

A study reported the outcomes of 19 patients treated with SBRT (45 Gy/6 fractions) for abdominal LNs oligometastases with 77.8% of local control at two years and minimal acute and chronic toxicities (Grade 3 in 1 patient). However, the prescription had to be downscaled by 10% to 20% in six of 19 cases to keep within dose/volume constraints [7].

For the presented case, the localization of the lesion could not be safely treated with another focal treatment. Non-MR-guided-RT SBRT would have required a lower prescription dose, potentially limiting treatment effectiveness, as proved by the necessity of five daily adaptive plans used for our patient. Indeed, even if a well-designed intensity-modulated-RT on a conventional accelerator would certainly lead to acceptable plan, MR-guided-RT, thanks to its soft tissue visualization, allows for treatment without invasive placement of fiducial markers which would have been necessary for cone beam CT to improve tumor volume targeting and gating. Moreover, normo-fractionated regimen would have required a biologically effective dose (BED) of 72 Gy which would have led to unacceptable toxicity. Actual management of oligometastatic disease uses SBRT as ablative treatment for an alternative to surgery radiotherapy due to its ability to deliver an ablative dose of radiation in a short treatment course with a higher biological effective dose. More importantly, MR-guided-RT enables more precise visualization of OAR in order to perform online adaptive RT and improves sparing of radiosensitive structures. Our current Tri-Cobalt-60 MRI-guided-RT platform is limited by dose gradients which impair the plan quality. This issue may be solved with the MR-Linac version.

## Conclusions

We report this case to demonstrate that SMART is an effective treatment modality for abdominal oligometastasis from breast cancer. Randomized trials are ongoing to evaluate the role of SBRT for oligometastases (NRG BR002, CORE trial). The emerging use of MR-guided-RT may play an important role in this field, allowing for a safe and effective treatment for challenging locations, such as the abdomen with ever-shifting internal anatomy.

## References

[1] Tree AC, Khoo VS, Eeles RA (2013). Stereotactic body radiotherapy for oligometastases. Lancet Oncol.

[2] Lewis SL, Porceddu S, Nakamura N (2017). Definitive stereotactic body radiotherapy (SBRT) for extracranial oligometastases: an international survey of >1000 radiation oncologists. Am J Clin Oncol.

[3] Mutic S, Dempsey JF (2014). The ViewRay system: magnetic resonance-guided and controlled radiotherapy. Semin Radiat Oncol.

[4] Hellman S, Weichselbaum RR (1995). Oligometastases. J Clin Oncol.

[5] Swain SM, Baselga J, Kim SB (2015). Pertuzumab, trastuzumab, and docetaxel in HER2-positive metastatic breast cancer. N Engl J Med.

[6] Henke L, Kashani R, Robinson C (2018). Phase I trial of stereotactic MR-guided online adaptive radiation therapy (SMART) for the treatment of oligometastatic or unresectable primary malignancies of the abdomen. Radiother Oncol.

[7] Bignardi M, Navarria P, Mancosu P (2011). Clinical outcome of hypofractionated stereotactic radiotherapy for abdominal lymph node metastases. Int J Radiat Oncol Biol Phys.

